# Anatomical study of the technique of the axis laminar screws and development of guide

**DOI:** 10.1186/s13018-023-03784-8

**Published:** 2023-05-05

**Authors:** Maji Sun, Fuchao Chu, Chunjiu Gao, Shuo Yang, Chen Long, Zhongwei Li, Gen Li, Jibin Wu, Feng Yuan

**Affiliations:** Department of Spine Surgery, The Affiliated Hospital of Xuzhou Medical University, Xuzhou, 221006 Jiangsu People’s Republic of China

**Keywords:** Axis, Laminar screws, Guide, 3D printing, Computer simulation

## Abstract

**Purpose:**

To develop a bidirectional slide guide to assist screw placement in the axial lamina and to preliminarily discuss the accuracy and feasibility of auxiliary screw placement.

**Methods:**

CT data from 40 randomly selected patients were imported into the software for modelling, and cross-pinning was used to simulate pinning. According to the different crossing methods of the upper and lower laminar screws, they are divided into two groups. In the software, the position of the needlepoint of each screw is accurately measured, and the needle point is kept unchanged to simulate the movable range of the screw tail under the condition that the body does not penetrate the cortical bone. The data were compared by grouping and gender. Finally, the guide was designed by combining the screw exit point and fine adjustment angle data of all patients with the centripetal principle of the slide rail.

**Results:**

The needle exit data L1/L2/L3/L4 were 6.44 ± 0.52 mm, 7.05 ± 0.48 mm, 3.55 ± 0.75 mm and 5.09 ± 0.74 mm, respectively, and the fine adjustment angle of the slide rail was 10.51° ± 0.87°. There was no significant difference between the two groups or between men and women (*p* > 0.05).

**Conclusion:**

In this experiment, using the data obtained from the simulation of screw insertion, a two-way slide guide was designed to assist the insertion of axial laminar screws. The guide locks the screw outlet point to position and guides the screw inlet point, which improves the accuracy and safety of screw placement.

## Introduction

In the clinic, spinal instability often occurs due to trauma, metastatic tumours, inheritance, infection, and other reasons. Atlantoaxial instability, which is located at the stress concentration of the head and neck, is the most common in the cervical vertebra, and this kind of instability often requires internal fixation surgery. The atlantoaxial position is deep, and there are important nerves and blood vessels nearby, which increases the risk and difficulty of internal fixation. At present, the main methods of posterior atlantoaxial fixation include articular process fixation, lateral mass fixation, and transpedicular internal fixation [1, 2]. Although the method of transarticular process fixation can ensure its strong biomechanical characteristics, it is difficult to achieve. According to related autopsy studies, approximately one-fifth of patients have variations, such as high suspension and bending of the vertebral artery, and C1/2 transarticular screws cannot be used safely [3]. In axial transpedicular fixation, the screw path needs to avoid the transverse foramen of C2 and at the same time avoid invading the spinal canal. However, the anatomical structure of the C2 pedicle, such as the angle and diameter of the pedicle, may have some variation, which leads to a greater risk of pedicle screw placement [4]. For special cases, such as when the pedicle screw is unsuitable for fixation due to the variation in the anatomical position of the pedicle, the width of the C2 pedicle isthmus is less than 3.5 mm, the position of the vertebral artery is abnormal, the bone is hypoplastic and the pedicle is small in volume, and C2 lamina crossing screw internal fixation technology has certain advantages. The anatomical structure of the axial vertebra is notably different from that of other cervical vertebrae, and its lamina thickness is larger, which lays a strong biomechanical foundation for the insertion of the axial laminar screw [5]. Wright [6] also confirmed this point through research, and lamina screws can avoid vertebral arteries, and their practicability has also been confirmed in the clinic [7]. In biomechanical tests, lamina screws can achieve the same effect as axial pedicle screw and transarticular screw fixation [8]. Clinical reports show that C2 lamina screw fixation technology is intuitive in operation and has advantages in safety. However, during the operation, most surgeons determine the screw entry point and angle with the naked eye, and the accuracy and safety of screw insertion will be influenced by the operator's experience, perspective effect, intraoperative posture, and other factors [9]. Auxiliary guide screw placement can diminish the above situation, reduce the risk and improve the efficiency of screw placement. However, most of the existing axial guides have the following disadvantages: (1) Individualized customization requires a long time to prepare before surgery, and the cost is high. (2) The guide plate should be close to the curved surface of the vertebrae during use, and the accuracy of screw placement is easily affected by soft tissue. (3) Polyethylene is used, and the hardness is insufficient, so it will be deformed during use. Based on the above problems, this study aims to design a guiding device with simple operation, stable structure, accurate guiding, safe use, and reusability. Based on the centripetal principle of the slide rail, the safety screw path is simulated by three-dimensional modelling technology, and the needle insertion point is simulated and debugged. Finally, the bidirectional slide rail guide of the axial laminar screw is designed.

## Methods and steps

### Three-dimensional model of the cervical spine and establishment of a safe screw path

In this experiment, 40 patients with 3D CT data were randomly selected, including 19 female patients and 21 male patients, with an average height of 172.9 ± 9.5 cm and an average age of 51.1 ± 12.8 years. Patients with axial lamina defects and hypoplasia were excluded.

**Fig. 1:**
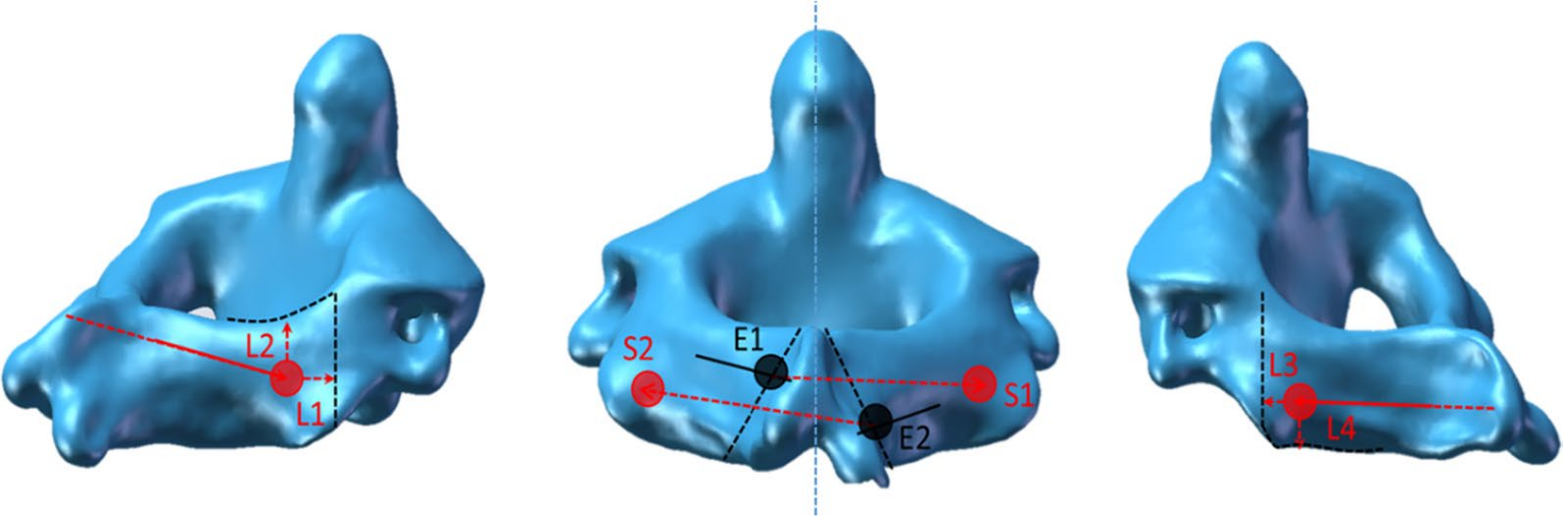
L1, L2, L3, and L4 indicate the measured distances, E1 and S1 are the upper lamina screw entry and exit points, and E2 and S2 are the lower laminar screw entry and exit points

**Fig. 2:**
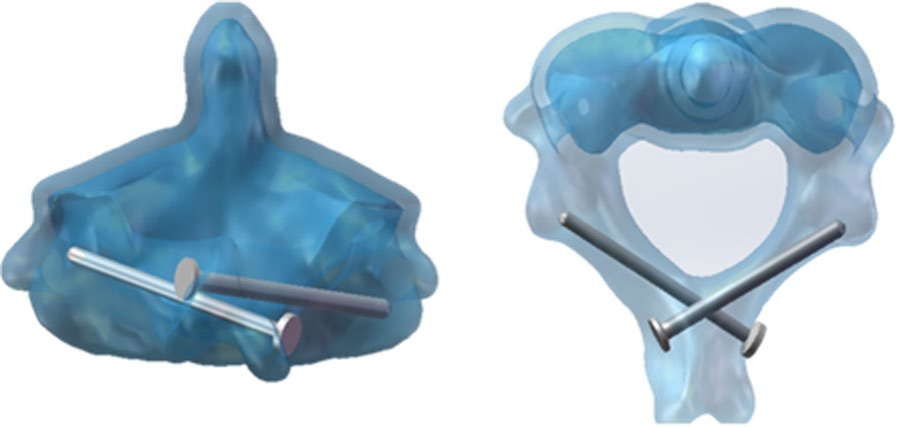
Schematic diagram of the intra-vertebral plate path of the screw

The cervical spine CT data of 40 patients were imported into mimics21.0 (Materialise, Belgium) software for 3D reconstruction. The reconstructed 3D model of the axis was imported into solidworks2021 (Dassault Systemes, America) for simulated screws insertion and distance and angle measurements. The screw insertion point and angle were selected by the standard screw insertion method to simulate screw insertion. (1) According to the up-and-down position of the laminar screw, it can be divided into upper laminar screws and lower laminar screws. (2) The upper lamina screw entry point is approximately 5 mm from the midline of the spinous process, and the lower laminar screw entry point is approximately 9 mm from the midline of the spinous process on the other side, all of which pass through the intersection of the lateral outer edge of the opposite lamina and articular process. Forty patients were randomly divided into two groups according to different directions of screw placement, with 20 patients in each group. In the first group, the superior laminar screw entered the left lamina, and the lateral edge of the right lamina passed out. The lower lamina screw passes through the right lamina and passes through the left lamina. The direction of screw placement in the second group was opposite to that in the first group. (3) The distance between the upper vertebral screw and the lateral edge of the vertebral plate is L1, the distance between the upper vertebral screw and the upper edge is L2, the distance between the lower vertebral screw and the lateral edge of the vertebral plate is L3, and the distance between the lower edge is L4 (Fig. 1). The distance measurement in the measuring tool was used to measure the length of L1, L2, L3 and L4, and then the two groups of data were compared (Fig. 2).

### Principle of centripetal slide guide

There are some differences in the anatomical structures of the different axes of patients, so the guide can maintain the accuracy of the needle insertion point. In this study, the slide rail design is added to the guide trunk, as shown. The principle of the slide rail is to adjust the needle entry point with the screw needle exit point as the centre of the circle to ensure that the guide sleeve always takes the needle exit point as the centre of the circle and points to the needle exit point during the adjustment process.

**Fig. 3:**
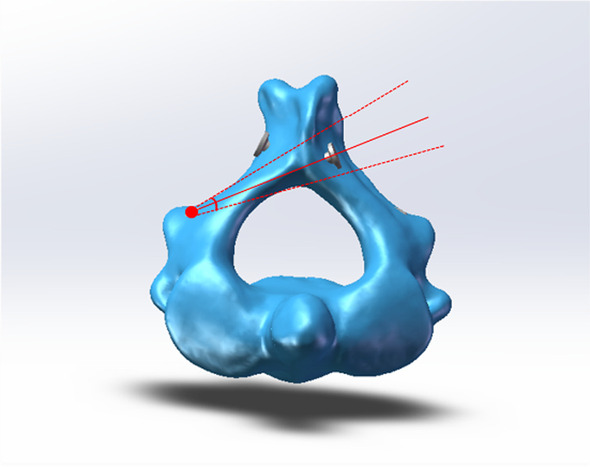
Fine angulation is obtained without the screw penetrating the medial and lateral cortical bone

The distal needle exit point was locked after the laminar screw was inserted into software. On the premise that the screw does not penetrate the medial and lateral cortex of the axis, the movable angle of the tail end of the laminar screw is simulated, and the fine-tuning angle is obtained (Fig. 3). The data obtained were compared by grouping and gender (Tables 1, 2).

**Table 1:** Comparison of the position of the screw-out point and the fine-tuning angle of the slide rail between the two groups ($\overline{x}$ ± *s*, *n* = 40)

Group	First group	Second group	*p*
L1 (mm)	6.55 ± 0.75	6.31 ± 0.61	> 0.05
L2 (mm)	7.12 ± 0.95	7.05 ± 0.89	> 0.05
L3 (mm)	4.06 ± 0.71	3.55 ± 0.64	> 0.05
L4 (mm)	4.97 ± 0.68	5.11 ± 0.76	> 0.05
Fine adjustment angle (°)	9.74° ± 0.88°	10.61° ± 0.79°	> 0.05

**Table 2:** Comparison of the position of the screw-out point and the fine-tuning angle of the slide rail between different genders (adopt the first group set of placement methods)

Group	Male	Female	*p*
L1 (mm)	6.71 ± 0.72	7.11 ± 0.73	> 0.05
L2 (mm)	7.19 ± 1.11	6.93 ± 0.84	> 0.05
L3 (mm)	3.75 ± 0.69	3.81 ± 0.80	> 0.05
L4 (mm)	5.15 ± 0.47	4.99 ± 0.86	> 0.05
Fine adjustment angle (°)	10.87° ± 0.74°	9.68° ± 0.91°	> 0.05

### Manufacture of a two-way slide guide for the axial laminar screw

Combined with the screw-out point data, the fine-tuning angle of the guide, and the centripetal principle of the slide rail, the three-dimensional model of the guide is modelled in the solidworks software. The main frame model of the guide is transmitted to an eosm400 laser metal 3D printer in STL format, and the main frame of the guide model is printed by selective laser melting technology. The model data of the guide sleeve and slide rail connecting ring are imported into a 3D printer (FDM-3000, Stratasys) and printed.

### Specific implementation method

**Fig. 4:**
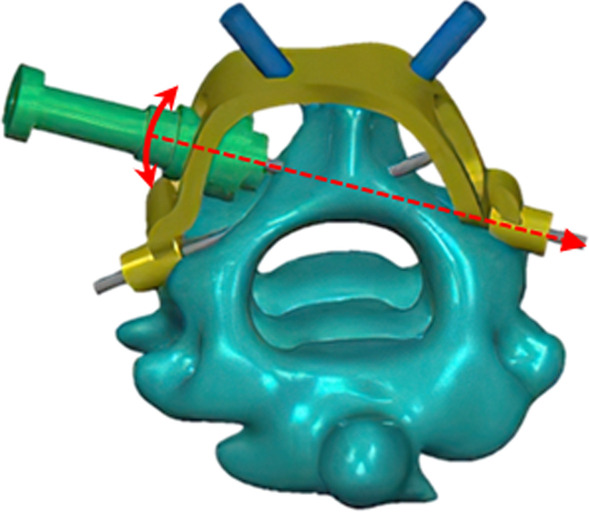
Yellow is the main body of the fixing frame; green is the sleeve of guide; blue is the spinous process locking sleeve

During the operation, the posterior arch of the atlas and the vertebral lamina of the axis are fully exposed, and the structure of the posterior part of the segment to be operated on is exposed. Then, the screw outlet socket of the guide is fixed at the intersection of the posterior edge of the vertebral lamina of the axis and articular process. The position of the guide sleeve of the slide rail is adjusted to find the ideal screw entry point. Then, the three-stage guide sleeve is locked at the screw entry point. After the position of the fixing frame is determined, Kirschner wires are inserted for fixation (Fig. 4). Then, the Kirschner wire is inserted into the sleeve of the third-stage guide, and the integrity of the ventral cortex of the vertebral lamina was explored by a ball probe. A hollow screw with a diameter of 3.5 mm and an appropriate length is inserted along the 2/3 stage sleeve and the Kirschner wire.

## Results

According to statistics, the positions L1/L2/L3/L4 of the total sample size are 6.44 ± 0.52 mm, 7.05 ± 0.48 mm, 3.55 ± 0.75 mm, and 5.09 ± 0.74 mm, respectively, and the fine adjustment angle of the slide rail is 10.51° ± 0.87°. In this experiment, based on the data obtained by measuring the total sample size and the centripetal principle of the arc slide rail, a two-way slide guide was designed to assist the insertion of the axial laminar screw. The guide device comprises a fixing frame main body, a three-stage sleeve, an arc-shaped centripetal slide rail, a slide rail connecting ring, and other parts. The upper end of the fixing frame body is a spinous process locking sleeve, which can penetrate Kirschner wire to fix the guide on the spinous process, and the middle part is an arc-shaped centripetal slide rail. The guide sleeve can be fixed inside the slide rail through the slide rail connecting ring to ensure the centripetal nature of the sleeve. At the same time, the guide sleeve has a three-level structure, which can complete all screw setting operations. To avoid the grinding and collision of cross-screws, the optimal angle of cross-screws is simulated in 3D. The main body of the guide is made of metal, which reduces deformation during operation and achieves better stability.

## Discussion

### Selection of the C2 fixing method

The atlanto-axis belongs to the structure of the upper cervical vertebra, and its area is deep. There are important nerves and blood vessels near the axis, so it is difficult to operate, and there is high risk of injury during the surgery. Currently, the following methods are commonly used for fixation of the axis screw, including lateral mass screws, C1/2 joint screws, C2/3 joint screws, and transpedicular fixation techniques. However, all of the above techniques have the risk of vertebral artery injury in clinical application. In 2004, Wright [10] proposed the technique of axial lamina cross-screw fixation to reduce the risk of injury in the vertebral arteries and nerves in the process of fixation. This technique makes full use of the advantages of the wide anatomical structure of the axial lamina and can avoid injury to arteries and nerves [11]. Its biomechanical stability is similar to that of axial pedicle screw placement. Through clinical research, a satisfactory fixation effect has been obtained. Through the observation of a large number of cadaver specimens, Cassinelli et al. [5] found that 70% of human axis vertebral plate thickness exceeded 5 mm, 92% exceeded 4 mm, and the average thickness was 5.7 mm. The average length of the practical screw track of the vertebral plate was 24 mm, and 99% of all specimens were over 20 mm in length, which indicated that it was feasible for most people to fix the vertebral plate screws in the axis lamina.

### Clinical significance of guidance assistance in placing screws in the C2 vertebral plate

Since C2 vertebral plate screw fixation technology was reported, it has been recognized by clinicians because of its simple operation and high safety. However, in clinical practice, most surgical operators completely rely on anatomical markers of the lamina to locate, and the accuracy of screw placement will be affected by factors such as operation experience, fluoroscopy effect, and intraoperative posture of surgical operators. At present, it has been reported that the direction deviation of screws during surgery breaks through the abdominal wall of the vertebral plate and enters the spinal canal, resulting in high spinal cord injury [12]. In addition, the vertebral artery may be damaged if the screw length or direction is not properly grasped during screw placement [13]. Therefore, Lehman et al. conducted many screw placement experiments and anatomical analyses on cadaver specimens to avoid screws entering the vertebral canal and damaging the vertebral artery. The study found that because the structure and screw track of the C2 lamina differ from those of the pedicle, it is difficult to confirm whether the screw deviates from the track or breaks through the inner wall and transverse process hole by conventional methods, and the accuracy of screw position based on conventional perspective images and naked eye observation is only 77.4%. Therefore, in the process of screw placement, we cannot rely on the auxiliary guidance of perspective to determine the position of the screw track. Multidimensional fluoroscopy is a real-time image navigation technology based on intraoperative X-ray or CT scanning images. It breaks through the shortcomings of traditional technology and is different from overreliance on the operator's experience or projected position. Multidimensional fluoroscopy relies on accurate preoperative marking and real-time multidimensional fluoroscopic images during the operation, and young operators can also obtain ideal and accurate screw placement positions. However, the navigation system also has some shortcomings, such as the high cost of the system, which leads to an increase in the operation cost. At the same time, similar three-dimensional navigation systems often need professional technicians to operate, which increases the complexity of the surgery, and most hospitals do not meet the conditions of configuration and application. Therefore, this study aimed to develop a guide device with simple operation, stable structure, accurate guidance, safe use, and reusability for clinical applications.

### Clinical application of existing guidance technology

Three-dimensional data simulation and synthesis technology are applied to orthopaedics. By extracting the three-dimensional CT data of the cervical vertebra and establishing a personalized auxiliary guide device, the accuracy of axial laminar screw placement can be improved to some extent. The personalized guide device is made by reconstructing the preoperative cervical spine image data to obtain a three-dimensional model. The manufacture of a personalized guide device can reconstruct the preoperative cervical spine image data of the patient to obtain a three-dimensional model. On the model, the structural data of the guide device are obtained by simulating the ideal screw track, and finally, it is achieved by processing and manufacturing special technologies such as three-dimensional printing. Three-dimensional printing process technology makes the three-dimensional model by spraying technology, which saves laser equipment in the process and can obtain faster printing speed and higher three-dimensional reduction degree. This process improves the printing efficiency and accuracy, reduces the printing cost, and controls the density of the printing model. Lu et al. [14] made a custom-made navigation template with photosensitive resin as the material. Analysis of the accuracy and clinical application of screw placement in cadavers showed that the customized navigation template based on this technology can increase the efficiency of screw implantation in the C2 lamina [15]. However, there remain some defects associated with this technology [16]; in the design process of the navigation template, more software is needed, and the original imaging data are easily lost in the conversion process, thus affecting the accuracy of the final modelling. Furthermore, because the material is resin, its thermal decomposition products have biological toxicity, and there are potential safety hazards in clinical application. Moreover, after moulding, it is easy to deform in use, resulting in guiding error.

At present, the existing axial guide uses RP (rapid prototyping) technology to make the physical model [17–19], but it needs to be customized in advance, which is expensive and takes a long time to prepare before an operation. At the same time, it must be closely attached to the vertebral plate to ensure accuracy. Once the soft tissue of the vertebral plate is cleaned improperly or the vertebral plate structure is damaged, the accuracy of the navigation will be affected. The insufficient hardness of polyethylene will lead to structural instability, and the error of 3D simulation and the shortage of fixed points will affect the accuracy of orientation.

### Advantages of bidirectional slide guide in assisting the placement of axial laminar screws

In the design of this guide, many factors, such as axial lamina deformity and fracture, are considered. Forty cases of 3D modelling samples are compared according to different screw placement methods and genders, and the position of screw exit points is measured. According to the obtained data, a slide guide with a wider application range is designed. Only the screw outlet interface contacts the outer edge of the vertebral plate, and it can guide accurately even if soft tissue remains or the vertebral plate structure is damaged. The guide is a titanium frame structure with high hardness and low deformation rate, which greatly improves structural stability. Two-way slide guidance does not require repeated fluoroscopy in navigation operations, which can greatly reduce the operation time and radiation dose. Because it can be reused, it is superior to the custom guide in cost. The guide can lock the screw entry point and the screw exit point of the lamina screw at the same time and then fix the guide on the spinous process by using two cross-fixing frames, which can lock the safe screw path and stabilize the guide, thus improving the double cortex rate of screw placement and ensuring the accuracy of screw placement. The entire operation can be completed visually, which has great clinical prospects. There are also shortcomings in this study, such as the limitation of sample size and lack of data measurement of bidirectional sliding guides in actual screw placement.

In summary, this guide is universal, and it has many advantages in the process of axis laminar screw placement, such as a stable main structure, firm multipoint fixation, accurate guidance, simple operation, and simultaneous placement of bilateral laminar screws. It greatly reduces the operation time, improves the operation efficiency, and provides an economical and reliable auxiliary method for the placement of axial laminar screws.

## Conclusion

According to the centripetal principle, locking the screw exit point and positioning and guiding the screw entry point can prevent the screw tip from going into the spinal canal due to the deviation in the insertion process. Two screw placement methods and data comparisons of different sexes ensure the universal applicability of this guide. With the help of spinous locking sleeves and titanium frames, the stability of screw placement is high, which improves the accuracy and safety of screw placement.

## Data Availability

The datasets used and/or analysed during the current study are available from the corresponding author on reasonable request. We do not have ethical permission to upload the data set into a repository. Please note that all study data has been anonymized for confidentiality purposes.
